# Need-based care of multi-morbid patients – supporting general practitioners with algorithm-generated recommendations of healthcare services (telemedicine-project ATMoSPHÄRE)

**DOI:** 10.1186/s12875-021-01537-2

**Published:** 2021-10-08

**Authors:** Peggy Borchers, Steve Piller, Mandy Böhme, Karen Voigt, Antje Bergmann

**Affiliations:** 1 Department of General Practice, Medical Clinic III, Faculty of Medicine Carl Gustav Carus, Technische Universität Dresden, Fetscherstraße 74, 01307 Dresden, Germany; 2 vital.services GmbH, Leipzig, Germany

**Keywords:** Telemedicine, Recommender system, Primary care, Multimorbidity, Decision support system, eHealth

## Abstract

**Background:**

The patient-oriented and need-based care of multi-morbid patients with healthcare services and assistive products can be a highly complex task for the general practitioners (GPs). An algorithm-based **digital recommendation system** (DRS) for healthcare services was developed within the context of the telemedicine research project ATMoSPHÄRE. The plausibility of the DRS was tested and the results used to examine if, and to what degree, the DRS provides useful assistance to GPs.

**Methods:**

The plausibility of the recommendations of the DRS were tested with the Delphi procedure (*n* = 8) and Interviews (*n* = 4) in collaboration with the GPs. They proposed services and assistive products they considered appropriate for two multi-morbid patients. Furthermore, GPs had to report whether, and to what degree they deemed the algorithm-generated recommendations appropriate. Significant quantitative differences between the GPs’, and the algorithm-generated, recommendations were evaluated with paired-samples-Wilcoxon-test.

**Results:**

The first Delphi round revealed a high variability regarding the amount and character of services recommended by the physicians (1 to 10 recommendations, mean = 5.6, sd = 2.8). These professional recommendations converged after consideration of the algorithm-generated recommendations. The number of algorithm-generated recommendations which were judged as appropriate ranged between 7 and 17 of a total of 20 (mean = 11.9, sd = 2.5). The interviews revealed that the additional algorithm-generated recommendations which were judged appropriate contained mainly social care services.

**Conlusion:**

The DRS provides GPs with additional appropriate recommendations for the need-based care of patients, which may not have been previously considered. It can therefore be assessed as a helpful complement in the primary care of multi-morbid patients.

**Supplementary Information:**

The online version contains supplementary material available at 10.1186/s12875-021-01537-2.

## Background

The increasingly growing quantity and complexity of available data has led to a renewed attention towards algorithms in IT Solutions and Systems [1]. Recommender systems are, depending on complexity and heterogeneity of necessary information, based on more or less complex algorithms. They aim to present selected, personalized content and offers to the user that could be of particular interest to them. They differ in the way they analyze data and their data sources. One way to generate recommendations is to compare the user data against the whole user database, identifying matches between the user and other users with similar behavior [2]. These recommender systems are primarily used by online eCommerce services such as Amazon and Netflix [2, 3].

In the context of healthcare, personal patient data and medical expertise serve as the main foundation for a patient-focused therapy and care solution. The progressing digitalization of medicine and healthcare means that much of this information is also available in digital formats. Using this data for assistance or recommender systems in clinical work should improve patient care. Recommender systems are increasingly emerging in the medical field and eHealth applications, for instance as algorithm-driven health service recommenders [4], clinical decision support systems [5] or for shared decision-making [6]. Recommender systems are now used to support the management of various diseases, such as heart disease [7] or diabetes [8] but also for example in smoking cessation [9].

The rising prevalence of chronic diseases amplifies the challenge that multimorbidity (multimorbidity defined as the concurrent presence of two or more chronic diseases) poses in the primary care of older patients [10]. Multimorbid patients constitute a very diverse group regarding disease combination, severity and treatment. The GP has to consider a multitude of information, often acquired in a long-lasting patient-doctor relationship.

Central treating targets of patient-centered care could be: involvement of the multimorbid patient in the prioritization of treatment targets (shared decision-making) [10], enabling the patient to remain at home, the preservation of patient functions, and the avoidance of hospital admissions. A multitude of options for treatment, consultation, and referral of support services exists to pursue these targets in primary care. The range of services aimed at improving and preserving independent living, in particular, continues to increase, driven by higher demand and new technological possibilities of recent years [11, 12].

This evolving diversity could be made available to the GPs by using patient-data-driven recommender systems to support the consultation in a way that is demand-oriented and also considers available care.

A digital recommendation system (DRS) was developed in the context of the joint telemedicine project ATMoSPHÄRE, employing an algorithm to match patients, according to their data, with appropriate health care services. Based on a plausibility check the appropriateness of the DRS was evaluated. The results were used to investigate the question of whether, and to what degree, the DRS can support the treatment of multimorbid patients in primary care.

## Methods

### Telemedicine project

The telemedicine project ATMoSPHÄRE started 2015 in Saxony and developed, employed, and evaluated a technology-based information and communication platform and a complementary home-based telemonitoring application. The aim of these tools was to improve the primary care of older, multi-morbid patients and therefore preserve and extend their ability to live independently. The telemedicine platform contained, in addition to the data provided by the telemonitoring application, information about socio-demographics, diagnoses, and therapy, as well as the results of various geriatric assessments. Detailed information about the study, including patient recruitment, study design, and additional information about the telemonitoring application developed for the patients, has been published elsewhere [13, 14].

### Digital recommendation system

The development and implementation of a digital recommendation system for external healthcare services by vital.services GmbH was a pivotal part in the second stage of ATMoSPHÄRE.

The DRS was intended to offer need-based, individual recommendations for medical and social care services to the study GPs, thereby assisting them in the planning of treatment.

**Fig. 1:**
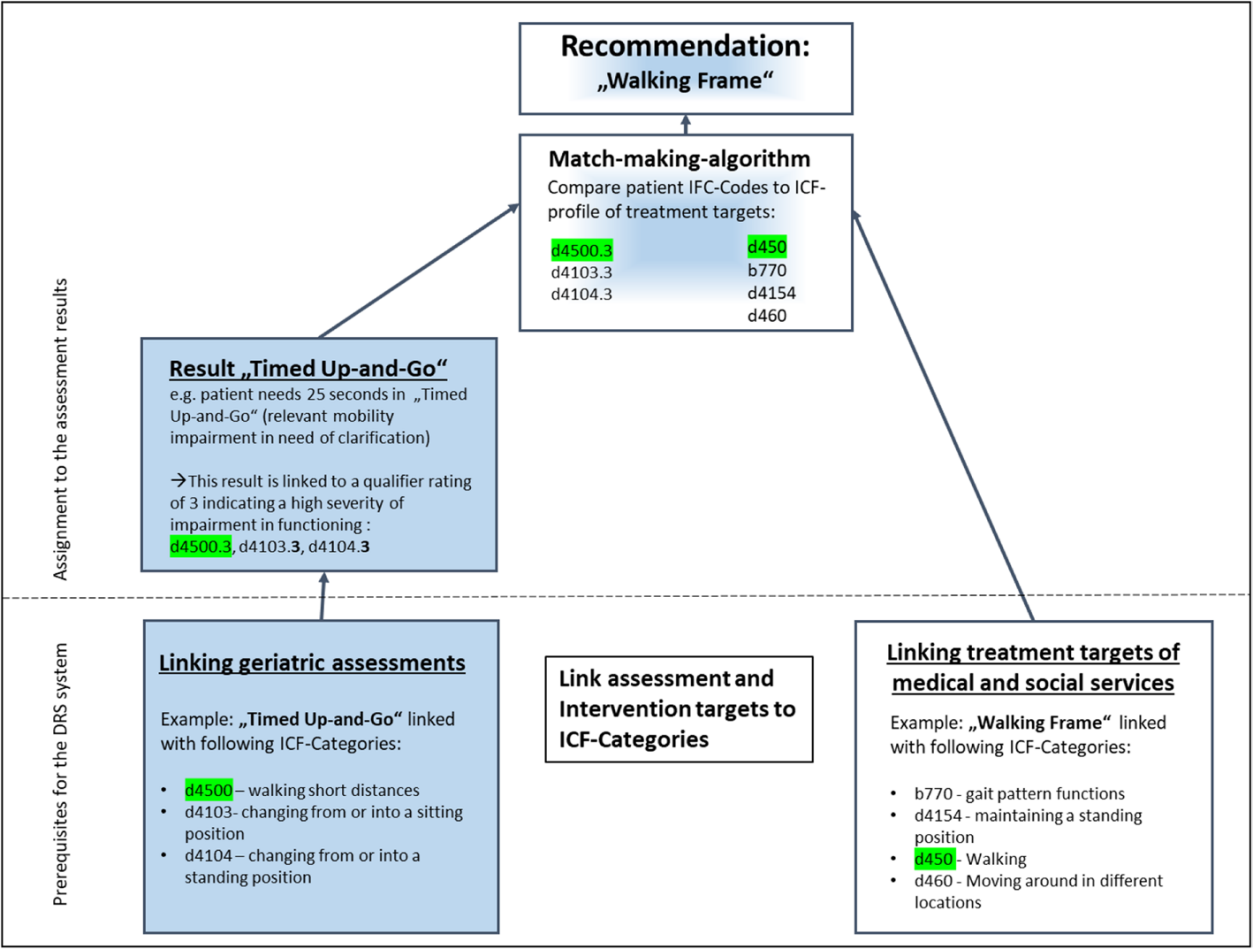
Schematic Diagram of the functional principle of the DRS, on the example of “Walking Frame”

Embedded into the telemedicine platform, the DRS used an algorithm to compare functional limitation of a patient with the treatment targets of a healthcare service (e.g. targets of occupational therapy), and determine how closely they matched. The DRS ranked all services according to how well they matched, and presented the closest matching services to the study GPs as recommendations on the telemedicine platform (Fig. 1).

The functional limitations were gathered using mostly commonly-employed geriatric assessments (Instrumental Activities of Daily Life (IADL) [15], Mini Mental State Examination (MMSE) [16], clock test [17], Timed Up-and-Go [18], ANGELINA [19], Geriatric Depression Scale (GDS) [20]). The treatment targets of services were taken or derived from different catalogues [21, 22]. Comparing functional limitations and treatment targets required to classify them within the framework of the International Classification of functioning, disability and health (ICF) [23]. Apart from providing a consistent and distinct vocabulary, the ICF possesses two additional properties utilized by the the DRS. Firstly, the mono-hierarchical structure allows definition of a proximity between categories. Secondly, the ICF-qualifier ratings signify the severity of disability within the corresponding category, which the DRS interprets as an indicator of need. The higher the severity of a functional limitation, the higher the need of a patient for a service which aims to alleviate that limitation.

Provided the limitations in functioning of a patient are classified within the ICF, each with the corresponding qualifier rating, and a set of healthcare services exists where the treatment targets of each service are converted into ICF-categories, a ranking of these healthcare services can be established. The treatment targets of a service or intervention are a set of ICF-categories representing the areas of functioning the service aims to support or improve. A service is considered more fitting the higher the qualifier rating of the function is, and the closer the function category and treatment target category are. An ICF category represents a specific function. The ICF qualifier indicates how severely the function is impaired. The qualifier ranges from 0 - meaning no impairment to 4 - indicating complete loss of function.

The functionality is shown schematically in Fig. 1 using the example of an impaired patient function indicated by the “Timed-Up-and-Go-Test” (relevant mobility impairment in need of clarification). The algorithm compares the ICF-coded assessment result of an individual patient to the ICF- categories representing the treatment targets of “walking frame”. For instance, the assessment result d4500.3 represents a severe impairment of the ability to walk short distances, this matches closely the treatment target d450 – Walking of the walking frame indicating this service could be appropriate. The closer the match and the higher the qualifier of the patient the higher the services will be ranked.

### Plausibility check

This publication bases on the results of the plausibility check of the DRS (Version 3.1.3) carried out by the Department of General Practice of the TU Dresden, which examined the adequacy of the DRS regarding its individually-compiled service recommendations for multi-morbid patients. This article is not intended to show the results of the entire plausibility check but rather presents the results of the first Delphi round and the interviews which entail the original answers and recommendations of the study GPs.

The operationalization of the adequacy of the DRS was achieved by a comparison with a determined reference value. The number and character of algorithm-generated recommendations were compared to the reference value regarding matching, missing, and additional fitting recommendations.

The reference value should represent the shared knowledge of a certain group, e.g. experts in a specific area [24]. In this context, the reference value is the set of the study GPs’ recommendations deemed appropriate for the treatment of a specific multimorbid patient.

The plausibility check followed a mixed-method approach and consisted of a) a two-staged Delphi procedure, evaluating the recommendations for two case vignettes (cv), and b) interviews with ATMoSPHÄRE study GPs, evaluating recommendations for selected study patients.

Based on a previous analysis of the most common supply needs of the study cohort, two profiles for multimorbidity were created. Patients corresponding to profile 1 all had indications for polypharmacy and were additionally diagnosed with type 2 diabetes mellitus and hypertension. Profile 2 patients showed irregularities in dementia-related assessments (MMSE or clock-test), or were already diagnosed with dementia. Using these two profiles, two corresponding study patients were selected and then utilized as anonymous case vignettes during the Delphi procedure. The data of these patients stand vicariously for the ailments and treatment issues patients of these profiles present during consultation hour. In the same way, two study patients were selected to be evaluated by study GPs for the evaluation via interviews.

### Data acquisition

**Fig. 2:**
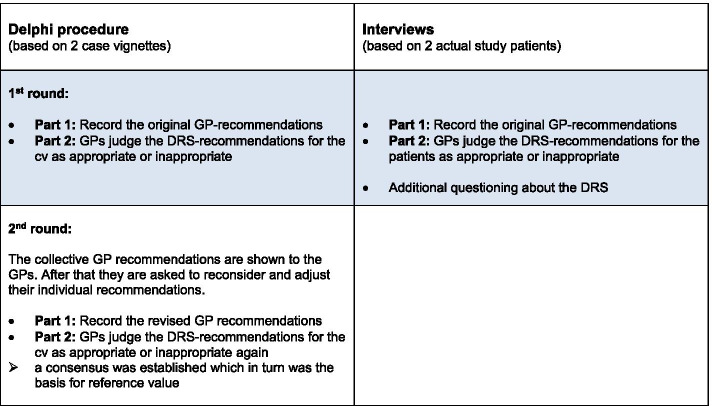
Illustration of the data collection (highlighted blue: results of this part is presented within this article)

The Delphi procedure was carried out in writing and consisted of two survey rounds. In both rounds the study GPs received a two-part questionnaire. The original GP- recommendations for the case vignettes (Part 1) as well as their approval or disapproval for the DRS-recommendations (Part 2) were collected in the first Delphi round. In the second round of the Delphi process the GPs were confronted with their own recommendations/answers and the recommendations of the other participating GPs and given the opportunity to reconsider decisions made in the 1st round (Fig. 2).

The purpose of the second round was to establish a consensus among the GPs which in turn was used to determine the reference value needed to evaluate the DRS-recommendations. This round is therefore the basis of the plausibility check but is not presented in this article.

In the first part of the questionnaire two case vignettes were introduced, including socio-demographic context, diagnoses, medication, and results of the geriatric assessments (see Fact sheet in Tables 1 and 2). The study GPs recommended healthcare interventions and services which they considered helpful for the case vignettes at hand. Part 1 was therefore used to determine the reference value. In Part 2 the recommendations of the DRS for the two case vignettes were presented to the study GPs. They were asked to judge which recommendations they considered as appropriate or inappropriate (see Additional file 1).

**Table 1:** Fact sheet and variability of types of recommendations made by the study GPs for case vignette no.1 (Data from 1st Delphi round Part 1)

**Fact sheet case vignette no.1:** male, 81 years, married, *12 diagnoses, **10 prescription drugs								
**Identified health problems by the geriatric assessments:**								
**Daily activities and coping with ailments** more than 5 prescription drugs, daily functions mildly limited by pain, does little shopping and needs assistance, needs assistance for housekeeping, degree of care, regularly receives support from friends and family								
**Mobility** according to the “Timed Up & Go” result of 14 s the patient is mildly impaired, possesses and uses a walker, considers himself severely limited in exercising moderately difficult tasks and climbing landings, needs help leaving home, uses public transportation only accompanied								
**Cognition and mood** has difficulties remembering things or names, considers his health status below average, according to a score of 7 pts. at the geriatric depression scale a depressive disorder is likely								
* diagnoses: **hypertension**, Type 2 diabetes mellitus with renal complications, obesity, aneurysm of the aorta abdominalis, cardiomyopathy, ischemic heart disease, atrial fibrillations and atrial flutter, long-term therapy with anticoagulants, pulmonary emphysema, chronic disorder of the lower pulmonary passages, renal insufficency **prescription drugs: Losartan, Colecalciferol, Xipamid, Phenprocoumon, Torasemid, Tamsulosin, Ezetimib, Allopurinol, Bisoprolol, Pantoprazol								
**Original recommendations by GPs (Part 1)**	**GP** **1**	**GP** **2**	**GP** **3**	**GP** **4**	**GP** **5**	**GP** **6**	**GP** **7**	**GP** **8**
Housekeeper / Home Assistant	x	x	x		x	x	x	x
Everyday Help / Everyday Companion	x	x			x	x		x
Occupational Therapy/ Memory Training		x	x	x	x	x	x	
Check of prescription drugs	x	x			x		x	
Nursing Service (mainly preparing medication)		x	x				x	x
Discuss medication regime with life partner		x					x	
Pick-up and Return Services		x						
Sports Group		x			x	x		
Physiotheraphy / Gait Training		x		x			x	
Personal Emergency Response System		x			x	x		
Respiration Therapy				x				
Support Services (public transportation)				x				
Local guidance office for the elderly				x				
Psychosocial Counselling					x			
Nutritional plan and counselling					x			
Assistive Products for Personal Hygiene					x		x	
Self-help Group						x		
Sociotherapy						x		
Walker/ Walker Training						x

**Table 2:** Fact sheet and variability of types of recommendations made by the study GPs for case vignette no.2 (Data from 1st Delphi round Part 1)

**Fact sheet case vignette no.2:** female, 89 years, living alone, *18 diagnoses, **7 prescription drugs								
**Identified health problems by the geriatric assessments:**								
**Daily activities and coping with ailments** more than 5 prescription drugs, regularly receives Support through familiy and friends								
**Mobility** a “Timed Up & Go”- Value of 25 s indicates limited mobility, employs forearm crutches considers himself severely limited in exercising moderately difficult tasks and climbing landings								
**Cognition and mood** a MMSE-Score of 25 points indicates **mild Dementia**, according to the “clock test” (3 Points) a more detailed examination of dementia should be considered, patient has difficulties remembering items and names, mentioned psychological problems								
**Senses** has difficulties hearing despite hearing aid								
*diagnoses: idiopathic gout, Hyperuricemia, osteoporosis, varices on the lower limbs, spondylosis, scoliosis, thyroid nodules, gastro-oesophageal reflux disease, hypertension, hypercholesterolemia, multi-nodular goiter, senility, Presbycusis, vertigo, fatty liver **prescription drugs: Allopurinol, Pantoprazole, Torasemide, Ramipril, Ibandronic acid, Biotin, Calcium+vitamin D3								
**Original recommendations by GPs (Part 1)**	**GP** **1**	**GP** **2**	**GP** **3**	**GP** **4**	**GP** **5**	**GP** **6**	**GP** **7**	**GP** **8**
Everyday Help / Everyday Companion	x					x		
Housekeeper / Home Assistant					x		x	x
Occupational Therapy		x	x	x	x	x	x	
Check of prescription drugs						x		
Nursing Service (mainly preparing medication)		x	x		x		x	x
Pill Box		x						
Walker/ Walker Training		x	x	x	x			x
Sports Group		x			x			
Physiotheraphy / Remedial Gymnastics		x			x		x	
Personal Emergency Response System		x			x	x	x	
Audiologist		x						
Assistive products for personal hygiene			x		x			
Mobile-Care Services (1–2 times weekly)							x	x
Meals on Wheels							x	
Mapping of available Support-network						x		
Local non-profit support services					x	x		

Additionally, two actual study patients (not case vignettes) representing the 2 profiles were discussed with their GP in the interviews in the same manner (Part 1 + Part 2). Furthermore, the study GPs were interviewed about the DRS in general. The interview questions for the plausibility check were developed based on the SPSS principle according to Helfferich [25]. The interviews were conducted by a trained researcher at GPs’ office.

### Analysis

The statistical analysis (with SPSS Version 25) of the quantitative data consisted, in addition to data description, of a comparison of means of the number of recommendations (non-parametric Wilcoxon Rank Sum Test for paired samples) between GPs and DRS. To determine the relevance of the results, the effect size according to Cohen was calculated. Effect sizes with values of d ≥ 0.8 are considered strong effects [26]. A significance level α equal to 5% was defined.

## Results

Of the 14 enlisted study GPs, 8 participated in the Delphi procedure (m = 2, f = 6; age (*n* = 6): mean = 50.7, sd = 7.8). They were working within the primary care system in the state of Saxony, with a minimum of 10 years of professional experience. Four of the 8 study GPs participated in the interviews, as they care for the study patients that represent the patient profiles.

### Delphi procedure

The vignette-specific listing of healthcare services and interventions by the study GPs showed a high variability regarding character and quantity of recommendations (Tables 1 and 2).

**Fig. 3:**
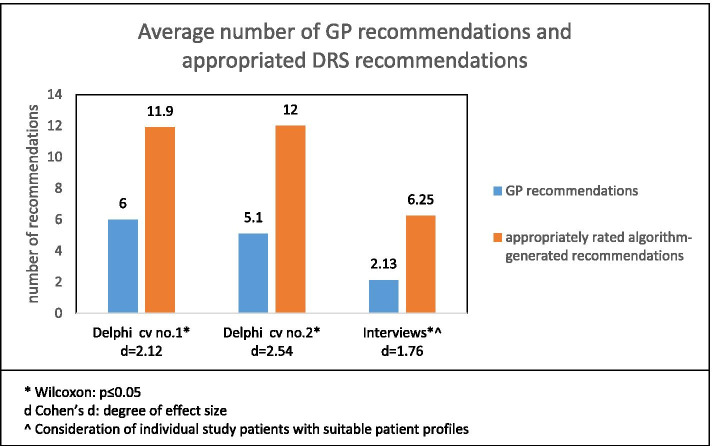
Comparison between the number of original GP recommendations and the number of algorithm-generated DRS recommendations rated by GPs as appropriate (Data from 1st Delphi round and interviews)

The study GPs indicated between three and ten (mean = 6, sd ± 2.9) recommendations for case vignette no. 1 (Table 1), and between one and nine (mean = 5.1; sd ± 2.9) for case vignette no. 2, respectively (Table 2). In comparison, the average number of appropriately rated algorithm-generated recommendations was significantly higher (asymptotic Wilcoxon-Test for both case vignettes: z = − 2.53, *p* = 0.011, *n* = 8), and roughly twice as many (case vignette no.1: mean = 11.9, sd ± 2.6; case vignette no.2: mean = 12, sd ± 2.6). The effect size according to Cohen was above 2.0 for both case vignettes (Fig. 3).

Examining the individual GP recommendations shows that only the first three recommendations for case vignette no.1 were made by a majority of physicians, meaning more than 4 of the 8 participating study GPs (Table 1). Likewise, only three recommendations were proposed by a majority for case vignette no. 2 (Table 2). After presentation of the algorithm-generated recommendations, the judgements converged (case vignette no.1: majority approval of 10 recommendations; case vignette no.2: majority approval of 12 recommendations) (Additional file 1).

### Interviews

A similar trend was found on conducting the interviews, where Part 1 consisted of the question: “Which prescriptions, healthcare interventions or services would you propose to your patient x?” Here, too, a significantly higher number of algorithm-generated recommendations were judged appropriate than previously listed by the study GPs for the patient (asymptotic Wilcoxon-Test: z = − 2.37, *p* = 0.018, *n* = 8). The effect size for the recommendations in the interviews was d = 1.76, which means a strong effect (Fig. 3).

Comparing the character of the recommendation categories shows that the DRS-recommendations additionally judged appropriate can be mainly assigned to social care (e.g. emergency-call service, pick-up and return services) and assistive products (e.g. pill dispensers). Occasional GP-made recommendations not included in the DRS pertain to special nursing services, or special assistive products, respectively.

All four interviewees confirmed in principle that the DRS is of added value to the GPs and the patient care. Requesting for specific assessment of the DRS prompted diverse responses. On one hand, the DRS was considered a helpful addition and reminder: “[...] *you might ask yourself: “Useful or not?”, but as a check or suggestion, that I do like*.[...] *I don’t always consider foot care if patients don’t have diabetes. That surely is something*.” (A1, f, 43) or: “*Well, it is useful to get a reminder. That’s alright, I guess*.” (A2, m, 44). On the other hand, the vagueness due to a lack of consideration of the patients’ priorities was criticized: “*It’s tough. Because I believe a lot of hard and soft data is missing for the digital recommendation system. The patient wishes for instance, the prioritisation, what is most important to the patient at that time*,[...].” (A3, f, 48).

## Discussion

The results of the first Delphi round representing the original answers of the study GPs show that despite having the same information available during the Delphi procedure, the study GPs’ recommendations varied heavily with respect to number and character. This points to the phenomenon that identical patient data can be interpreted very differently, subsequently leading to diverse expectations of patient needs, or that GPs may derive very different priorities respectively. Previous experience and methods, but also a lack of knowledge about (new) healthcare interventions and services, might explain the differing responses from the GPs.

In Part 2 the study GPs judged the DRS-recommendations according to their appropriateness and suitability for the case vignettes/actual study patients.

The study GPs judged a higher number of DRS recommendations appropriate, than they themselves made initially, for the case vignettes in the Delphi procedure, and study patients in the interviews, alike. The study GPs considered the algorithm-generated recommendations as a helpful complement or reminder of services. The recommendations reminded the study GPs of services which are rarely considered, or even forgotten, in day-to-day business. The recommendations deemed additionally appropriate pertained mainly to social care and assistive products.

However, the DRS failed to generate recommendations of some services which the study GPs considered necessary (e.g. “assistive products for personal hygiene” at cv no.1). This might be because the DRS could not match the patients’ limitation of functioning with the treatment target of the services in question, or the services were not available to the system because no reliable source was found which either had the treatment targets of these services already linked to ICF-categories or allowed for verifiable linking to the ICF.

The analysis shows potential for further optimization: the addition of more data for the DRS to evaluate, specifically the wishes and priorities of the patient, which cannot be derived from the geriatric assessments. Patient priorities should be considered in the therapeutic goals and in decisionmaking. Furthermore, incorporating the patients’ priorities increases trust and adherence [27].

### Strengths and limitations

As shown in other studies regarding the employment of assessments, geriatric assessments of multimorbid patients are only the basis for shared decision-making of therapeutic goals and healthcare decisions [28–30].

The enduring participation of GPs during the plausibility check testifies to the interest of physicians in this subject. Due to the small sample size and restriction to patient profiles in the plausibility check, the results cannot be generalised. A heightened interest into telehealth applications by the participating GPs can be assumed, leading to a likely selection bias. This might have intensified the positive judgement of a digital tool.

## Conclusions

The “patient-data-driven DRS of healthcare services” was developed as an algorithm-based recommender system in the context of the telemedicine project ATMoSPHÄRE, to provide GPs with patient-related and need-based proposals of healthcare services, with the goal to support them in treatment and healthcare decisions. The results of this study show that the DRS can support GPs treating multimorbid patients, by generating additional, potentially-helpful suggestions, which are unknown to, or not considered by the GP, but which may aid the patient in maintaining their functions and improve their quality of life.

A DRS can replace neither the doctor-patient-relationship, nor the joint prioritisation of therapeutic goals. The additional recommendations may, however, facilitate the dialogue between doctor and patient, and support patient-centered need-based care, which takes current healthcare services into account.

The identified potential for optimisation can be used to develop the system further. For instance, the ability to prioritise individual treatment targets according to the wishes of the patient as well as the addition of more services into the DRS-database would be beneficial. Regardless of the match-making algorithm, the system could benefit from a feature showing relevant healthcare service providers in proximity of the patient. Providing this information to the patient, by using the telemedicine application, could facilitate therapy planning.

## Supplementary Information

**Additional file 1: Appendix 1.** DRS-recommendations for cv no.1 judged appropriate by study GPs (Data from 1^st^ Delphi round Part 2). **Appendix 2.** DRS-recommendations for cv no.2 judged appropriate by study GPs (Data from 1^st^ Delphi round Part 2).

## Data Availability

The datasets used and analysed during the current study are available from the corresponding author PB on reasonable request.

## Abbreviations

cv: Case vingnette
DRS: Digital recommendation system
GDS: Geriatric Depression Scale
GP: General Practicioner(s)
IADL: Instrumental Activities of Daily Life
ICF: International Classification of functioning, disability and health
MMST: Mini Mental State Examination
