# Supplementary material for: Need-based care of multi-morbid patients – supporting general practitioners with algorithm-generated recommendations of healthcare services (telemedicine-project ATMoSPHÄRE)
Source: BMC Fam Pract. 2021 Oct 8;22:198. doi: 10.1186/s12875-021-01537-2 (PMC8499564; doi:10.1186/s12875-021-01537-2)
Supplement: Supplementary file 1 — Additional file 1: Appendix 1. DRS-recommendations for cv no.1 judged appropriate by study GPs (Data from 1st Delphi round Part 2). Appendix 2. DRS-recommendations for cv no.2 judged appropriate by study GPs (Data from 1st Delphi round Part 2). [file 12875_2021_1537_MOESM1_ESM.docx]

**Appendix:**

| **DRS-Recommendations for cv no.1 judged appropriate by study GPs** | **GP**  **1** | **GP**  **2** | **GP**  **3** | **GP**  **4** | **GP**  **5** | **GP**  **6** | **GP**  **7** | **GP**  **8** |
| --- | --- | --- | --- | --- | --- | --- | --- | --- |
| Motor-Functional Treatment | x | x |  | x |  | x | x |  |
| Sensorimotor-Perceptual Treatment | x | x |  |  |  | x | x |  |
| Ergotherapeutic brain conduction training/Neurophysiological oriented treatment |  | x | x |  |  | x | x |  |
| Long-term outpatient care | x |  |  |  | x | x |  | x |
| Mental-functional Treatment |  | x |  |  | x | x | x |  |
| Occupational therapy temporary splint | x |  |  |  |  | x | x |  |
| Understandable medication plan and review of long term medication | x | x | x | x | x | x | x | x |
| Consultation medication plan - annually | x | x | x | x | x | x |  |  |
| Consultation medication plan with pharmacist | x |  |  | x | x | x |  |  |
| Physiotherapy –group therapy |  | x | x |  | x | x | x | x |
| Physiotherapy – individual therapy |  | x |  | x |  |  |  | x |
| Physiotherapy – exercise bath |  |  |  |  |  |  |  |  |
| Physiotherapy – central nervous system |  |  |  |  |  |  | x | x |
| Visiting services | x |  | x | x | x | x | x | x |
| Personal Emergency Response System | x | x | x |  | x | x | x | x |
| Accompanied Travel |  |  |  |  | x | x |  | x |
| Support Services (public transportation) | x | x |  | x | x | x |  | x |
| Shopping and courier services | x | x | x | x | x | x |  | x |
| Housekeeper / Home Assistant | x |  |  | x | x | x | x | x |
| Pill Box | x |  | x | x | x | x | x |  |

*Appendix 1: DRS-recommendations for* ***cv no.1*** *judged appropriate by study GPs (Data from 1^st^ Delphi round Part 2)*

| **DRS-Recommendations for cv no.2 judged appropriate by study GPs** | **GP**  **1** | **GP**  **2** | **GP**  **3** | **GP**  **4** | **GP**  **5** | **GP**  **6** | **GP**  **7** | **GP**  **8** |
| --- | --- | --- | --- | --- | --- | --- | --- | --- |
| Ergotherapeutic brain conduction training/Neurophysiological oriented treatment | x | x | x | x | x | x | x | x |
| Sensorimotor-Perceptual Treatment | x |  |  |  |  | x | x |  |
| Understandable medication plan and review of long term medication | x | x | x | x | x | x | x | x |
| Consultation Medication Plan - annually | x | x | x | x | x | x |  |  |
| Consultation medication plan with pharmacist | x |  |  | x | x | x |  |  |
| Speech Therapy |  |  |  |  |  |  |  |  |
| Psychological - Functional Treatment | x | x |  |  | x | x | x | x |
| Physiotherapy –group therapy |  |  |  |  | x |  |  | x |
| Physiotherapy – individual therapy |  | x |  | x |  |  |  | x |
| Physiotherapy – exercise bath |  |  |  |  | x |  |  |  |
| Physiotherapy – central nervous system |  |  |  |  |  |  | x |  |
| Occupational therapy temporary splint |  |  |  |  | x |  |  | x |
| Visiting services | x | x | x | x | x | x | x | x |
| Personal Emergency Response System | x | x | x | x | x | x | x | x |
| Accompanied Travel | x |  |  | x |  | x | x | x |
| Support Services (public transportation) |  | x |  | x | x | x | x | x |
| Shopping and courier services |  | x | x | x | x | x | x | x |
| Business services at home - Hairdresser |  |  |  | x | x | x | x | x |
| Business services at home - Chiropody |  | x |  | x | x | x | x | x |
| Pill Box | x | x | x | x | x | x | x |  |

*Appendix 2: DRS-recommendations for* ***cv no.2*** *judged appropriate by study GPs (Data from 1^st^ Delphi round Part 2)*
